# Current Research on Flavor Compounds in Fermented Food Products

**DOI:** 10.3390/foods13050730

**Published:** 2024-02-28

**Authors:** Niël van Wyk

**Affiliations:** 1 Department of Microbiology and Biochemistry, Hochschule Geisenheim University, Von-Lade-Strasse 1, 65366 Geisenheim, Germany; nvw@brain-biotech.com; 2 ARC Centre of Excellence in Synthetic Biology, Department of Molecular Sciences, Macquarie University, Sydney, NSW 2113, Australia

Recent advancements in the field of food science have spurred a surge of research focused on unraveling the intricate world of flavor compounds in fermented food products [1,2,3]. Fermentation, a traditional food preservation method, not only enhances the shelf life and nutritional value of foods but also imparts unique and complex flavors. Current research delves into the identification, characterization, and understanding of the diverse array of flavor compounds produced during the fermentation process. Scientists are employing cutting-edge analytical techniques to dissect the volatile and non-volatile components that contribute to the distinct sensory profiles of fermented foods. Additionally, there is a growing interest in exploring the impact of microbial communities on flavor development, considering the pivotal role of microorganisms in fermentation [4,5,6,7]. This multifaceted approach not only enhances our knowledge of the chemistry behind fermented flavors but also holds promise for the development of novel and improved fermented food products with enhanced sensory attributes.

Fermentation is a biochemical process wherein microorganisms such as bacteria, yeast, or fungi metabolize carbohydrates, proteins, and fats, leading to the production of various metabolites, including flavor compounds. These compounds contribute to the aroma, taste, and overall sensory perception of fermented foods. The flavor profile of fermented products is influenced by factors such as the type of microorganism involved, fermentation conditions (e.g., temperature, pH, and duration), and the composition of the substrate.

Recent advancements in analytical techniques, such as gas chromatography–mass spectrometry (GC–MS) and high-performance liquid chromatography (HPLC), have enabled researchers to identify and quantify a wide range of flavor compounds in fermented foods [8,9,10]. Previous studies have revealed the presence of volatile organic compounds (VOCs), including alcohols, esters, acids, ketones, and sulfur-containing compounds, which contribute to the characteristic aromas and tastes of fermented products.

While traditional fermented foods continue to be celebrated for their cultural significance and culinary appeal [11,12,13,14], there is growing interest in exploring novel fermentation processes and substrates to create innovative flavor profiles. Researchers are currently investigating the use of unconventional microorganisms to ferment a diverse range of raw materials, including fruits, vegetables, grains, and legumes [15,16]. These endeavors have led to the development of novel fermented products with unique flavors and enhanced nutritional properties.

Furthermore, the application of biotechnological approaches, such as metabolic engineering and enzyme technology, holds promise for modulating the flavor profile of fermented foods [17,18,19,20]. By manipulating the metabolic pathways of fermentative microorganisms, researchers can tailor the production of specific flavor compounds, thereby fine-tuning the sensory characteristics of fermented products. Such advancements pave the way for personalized flavor preferences and the creation of niche market offerings in the food industry.

In addition to their culinary appeal, fermented foods are increasingly recognized for their potential health benefits and role in promoting gut health [21,22,23,24,25,26,27,28,29,30]. The fermentation process enhances the bioavailability of nutrients, increases the diversity of microbial species in the gut microbiota, and produces bioactive compounds with antioxidant and anti-inflammatory properties. The consumption of fermented foods has been associated with various health outcomes, including improved digestion, immune function, and metabolic health.

Furthermore, fermenting food materials offers opportunities for reducing food waste and promoting sustainability in the food supply chain [31,32,33,34,35]. Fermentation can extend the shelf lives of perishable ingredients, thereby reducing losses and contributing to the utilization of surplus produce. Additionally, the use of fermentation in food processing can mitigate environmental impacts by reducing energy consumption and greenhouse gas emissions compared to conventional preservation methods.

With this special edition, we aimed to attract contributions within this field and secured five original papers and one review. Below are short summaries of these contributions.

Coelho et al. explored the use of *Gentiana lutea* rhizomes commonly employed as a bittering agent in food for flavoring goat cheese production. Gentian-flavored goat cheeses were created by immersing unflavored goat cheeses into gentian-infused whey. Chemical and sensory analyses revealed that gentian-flavored cheeses exhibited reduced unpleasant notes, higher bitterness levels attributed to loganic acid transfer, and more complex flavor profiles compared to unflavored cheeses, with compounds such as 3-methyl butanoic acid and phenethyl acetate distinguishing gentian’s origin. This study suggested that gentian rhizomes hold promise as a flavoring agent in enhancing the sensory experience and reducing goaty perceptions in goat cheeses [36].

Xia et al. provided an extensive composition profile of wine made from sea buckthorn along with its distilled liquor, both known for their potential health benefits but hindered by unpleasant flavors. Through analyzing differential metabolites in the distillate of sea buckthorn, 133 volatile organic compounds were identified, with 22 contributing to its aroma. Fermentation notably enhanced the content of the volatile components, particularly esters, while distillation further increased the concentration of 51 volatile components. Positive correlations were found between sensor values and increased levels of alcohols and esters, reflecting trends in key components, offering insights into improving the flavor of sea buckthorn beverages [37].

Romano et al. wrote a comprehensive review that delved into the intricate relationship between grape fermentation and the aromatic complexity of wine, influenced by various factors, such as grape cultivars, ripeness, climate fermentation techniques, and microbial interaction. Yeast plays a crucial role in shaping the wine’s aroma, contributing to the synthesis of specific metabolites and the modification of many compounds found in grape must. Key aroma compounds with non-*Saccharomyces* yeast species are particularly noted for enhancing wine sensory profiles through enzymatic activities [38].

Baleiras-Couto et al. investigated the spontaneous fermentation of *Arbutus unedo* L. fruit, commonly used in spirit production, from two producers in central Portugal. Despite a diverse range of indigenous yeasts, *S. cerevisiae* isolates formed distinct clusters associated with each producer, influencing the physical–chemical and volatile compositions of the resulting distillates, thereby enabling differentiation between producers. While the harvesting period’s impact was less pronounced, the characterization of indigenous yeasts offers valuable insights for preserving the unique qualities of *Arbutus unedo* L. fruit distillates [39].

Li et al. explored the impact of microbial inoculation with *Tetragenococcus halophilus* and *Wickerhamomyces anomalus* on the physicochemical and flavor properties of soy sauce during early-stage moromi fermentation at 22 °C. Their results indicated that single yeast or LAB inoculation enhances amino nitrogen, lactic acid, acetic acid, free amino acid, and key flavor component production levels. The sequential inoculation of *T. halophilus* and *W. anomalus* yielded higher levels of free amino acids and aromatic compounds, potentially due to synergistic effects, resulting in characteristic soy sauce flavor compounds and improved savory, roasted, and caramel intensities, suggesting a promising approach for high-quality moromi production at lower temperatures [40].

Finally, Torres-Rochera et al. investigated the molecular interactions between salivary mucins and various wine phenolic compounds, including catechin, epicatechin, and quercetin 3-*β*-glucopyranoside, as well as the impact of the anthocyanin malvidin 3-*O*-glucoside on these interactions. Their isothermal titration calorimetry results revealed that anthocyanin demonstrates a stronger interaction with mucins compared to flavanols, with their affinity constant values notably higher for anthocyanin. Interestingly, flavonols appear to have minimal involvement in these interactions at typical wine polyphenol concentrations. These findings underscore the significance of wine anthocyanins in astringency mechanisms, particularly regarding high-molecular-weight salivary proteins, such as mucins [41].

The exploration of flavor compounds in fermented food products represents an interdisciplinary endeavor that encompasses scientific inquiry, culinary innovation, and societal impact. Current research endeavors are unraveling the intricate biochemical pathways that govern flavor formation during fermentation, providing insights that transcend traditional culinary practices. From deciphering the molecular basis of flavor perception to harnessing biotechnological tools for flavor modulation, the study of fermented foods continues to captivate researchers and food enthusiasts alike. As we delve deeper into the realm of flavor complexity, the potential for culinary creativity, health promotion, and sustainability in fermented foods remains ripe for exploration.
